# The specific nature of the statistical learning deficit in developmental dyslexia: Impaired temporal and spared spatial learning abilities

**DOI:** 10.3758/s13423-026-02980-x

**Published:** 2026-09-15

**Authors:** Areen Mazzawi Shukri, Bethany Growns, Noam Siegelman, Yafit Gabay

**Affiliations:** 1 Department of Special Education and the Edmond J. Safra Brain Research Center for the Study of Learning Disabilities, University of Haifa, Mount Carmel, Haifa, Israel; 2 Department of Psychology and Department of Cognitive and Brain Sciences, Hebrew University of Jerusalem, Jerusalem, Israel; 3 The School of Psychological Sciences, University of Canterbury, Sydney, Australia

**Keywords:** Developmental dyslexia, Statistical learning, Conditional statistics, Distributional statistics, Non-spatial vs. spatial relations

## Abstract

While extensive research highlights the importance of statistical learning in language acquisition and processing, its relationship to developmental dyslexia, a common developmental language disorder, remains unclear. Studies of statistical learning in dyslexia often rely on specific tasks that most likely do not cover the scope of statistical learning as a multi-componential construct, making it difficult to discover what aspects of learning are affected in dyslexia. The present study provides the first systematic examination of two major facets of statistical learning in dyslexia. We investigated statistical learning performance among individuals with and without dyslexia using a 2 × 2 factorial design that systematically manipulated both the type of statistical relation between elements (non-spatial vs. spatial) and the structure of statistical input (conditional vs. distributional). Neurotypical participants demonstrated learning across all tasks, whereas individuals with dyslexia showed significantly reduced performance when statistical regularities involved non-spatial relations, though not when they involved spatial relations. This deficit was apparent regardless of whether tasks involved conditional or distributional statistics. The findings suggest that rather than being generally weak in statistical learning, individuals with dyslexia face challenges in learning environments involving non-spatial statistical regularities that require integration of statistical computations across multiple exposures. Given the involvement of non-spatial aspects in printed and spoken language, these deficits can add to the challenges faced by individuals with dyslexia in acquiring linguistic representations. Additionally, the dissociation observed in dyslexia supports the notion that statistical learning comprises distinct sub-processes rather than a single unitary process.

**Supplementary Information:**

The online version contains supplementary material available at https://doi.org/10.3758/s13423-026-02980-x.

## Introduction

Developmental dyslexia is a neurodevelopmental disorder that falls within the broader domain of language-based learning disabilities (Pennington & Bishop, 2009; Peterson & Pennington, 2022). Dyslexia is characterized by persistent difficulties with accurate and fluent word recognition, phonological decoding, and spelling, despite typical intelligence and exposure to appropriate educational opportunities. In dyslexia, the core impairment is in basic literacy skills such as reading accuracy, reading fluency, and/or spelling (Pennington et al., 2019), whereas in other language disorders, such as developmental language disorder (DLD), broader domains of language, including semantics, syntax, and pragmatics, are affected (Georgiou & Theodorou, 2023). Whereas traditional accounts assumed that neurodevelopmental disorders (NDDs) such as dyslexia and DLD are characterized by a deficit in one domain, more recent views propose that NDDs are likely to arise from multiple risk factors (for a review, see Kouki et al., 2025). One possible domain that has been increasingly linked to dyslexia in recent years is statistical learning (Lee et al., 2022; Singh & Conway, 2021).

### Statistical learning computations and their possible role in spoken and written language

Statistical learning is a fundamental cognitive mechanism through which organisms learn different types of structured regularities in their environment, across time and space, which are then used to guide behavior (Armstrong et al., 2017; Aslin, 2017; Conway, 2020; Conway et al., 2025; Frost et al., 2019; Orpella et al., 2021; Sherman et al., 2020). This mechanism is thought to play a significant role in many cognitive processes and particularly in spoken and written language acquisition and processing (Aslin, 2017). Recent frameworks of statistical learning propose that it likely encompasses multiple and distinct systems that adapt to varying informational demands, highlighting its multi-faceted nature (Arciuli, 2017; Bogaerts et al., 2022; Frost et al., 2015; Siegelman et al., 2017a, 2017b). Our work builds on this perspective by focusing on two dimensions: (1) the *type of statistical relation* between elements, specifically whether learned regularities are spatial or non-spatial; and 2) the *structure of statistical input:* whether learned regularities are conditional or distributional*. Spatial* statistical learning involves detecting regularities in spatial configurations, whereas *non-spatial* statistical learning involves extracting structured information that is not organized in space. *Conditional* statistical learning involves recognizing predictive relationships between sequential elements, while *distributional* statistical learning pertains to sensitivity to the frequency and variability of elements within a distribution. Research indicates that these forms of statistical learning may engage different types of computations, as evidenced by patterns of correlations across different statistical learning tasks, dual task designs, and theoretical models of statistical learning (Gabay et al., 2023a, b; Growns et al., 2020; Jiménez & Vázquez, 2011; Kidd et al., 2023; Siegelman & Frost, 2015; Thiessen et al., 2013; Zhou et al. 2024).

Considering statistical learning performance across these dimensions is important because different component skills of language and literacy may differentially depend on them (Siegelman, 2020). In the current work, we focus on statistical learning computations among individuals with and without dyslexia. We contend that literacy skills potentially draw on both non-spatial and spatial statistical learning computations, whether conditional or distributional. For example, non-spatial conditional statistical learning may influence processes pertaining to identification of letters and letter chunks that occur regardless of letter position (Fernández-López & Perea, 2023; Schoonbaert & Grainger, 2004). In parallel, spatial conditional statistical learning can help learners detect conditional probabilities (p(B|A) among sequences of letters within words (e.g., bigram frequency, Chetail, 2017; co-occurrences of letters forming affixes, Lelonkiewicz et al., 2020; and other “horizontal” statistics, Siegelman et al., 2025) and between words (e.g., word predictability, Conway et al., 2010). *Distributional* statistical learning entails learning the distribution of printed units in the language (p(A) vs. p(B)), both *spatially* (e.g., which letters or letter clusters are more or less frequent in a given position within words) and non-spatially, that is, distributions that hold regardless of spatial location (e.g., learning about the frequency distributions of letters, words, and multi-word units). Studies supporting this link between statistical learning, reading, and language reveal that success in non-spatial conditional statistical learning tasks (e.g., triplet task) correlates with stronger linguistic skills among children and adults (Arciuli & Simpson, 2012; Frost et al., 2013; Qi et al., 2019; von Koss Torkildsen et al., 2019) and further predicts subsequent language development (Monaghan et al., 2024; Ren et al., 2023). Similarly, performance on non-spatial distributional statistical learning paradigms (e.g., cross-situational statistical learning) has been linked to individual differences in language skills (Hu, 2017; McGregor et al., 2013, 2022; Penaloza et al., 2017). Findings for spatial conditional and distributional statistical learning tasks (e.g., serial reaction time tasks, visual artificial grammar learning tasks,) vary more, with some studies reporting reliable associations (Kidd, 2012; van der Kleij et al., 2019) and others failing to find such relationships (Schmalz et al., 2019; West et al., 2018). Meta-analysis evidence likewise remains inconclusive, reflecting the ongoing debate regarding the association between statistical learning computations and linguistic skills (Boeve et al., 2024; Lee et al., 2022; Ren et al., 2023).

### Statistical learning computations in developmental dyslexia

Statistical learning difficulties have been associated with dyslexia (Ballan et al., 2022; Bettoni et al., 2024; Dobó et al., 2021; Gabay et al., 2012; Gabay et al., 2015a, b; Howard Jr. et al., 2006; Kahta & Schiff, 2019; Kligler & Gabay, 2023a; Kligler et al., 2023; Lum et al., 2013; Mateus-Moreno et al., 2025; Menghini et al., 2006; Ozernov-Palchik et al., 2023; Pavlidou et al., 2010; Pavlidou & Williams, 2014; Pavlidou et al., 2009; Ringer et al., 2025; Sigurdardottir et al., 2017; Singh et al., 2018; Stoodley et al., 2006; Tong et al., 2019; Vandermosten et al., 2019; Vicari et al., 2005; Vicari et al., 2003). Indeed, a recent meta-analysis points to a reliable deficit in statistical learning among individuals with dyslexia (Lee et al., 2022). Nevertheless, most studies have not attempted to tie dyslexia to deficits in *specific types* of statistical learning computations. Rather, they have examined whether individuals with dyslexia have a “general” deficit, as observed in one or a small number of tasks (often arbitrarily chosen for a given study; see Bogaerts et al., 2021). Consequently, the precise nature of the relationship between dyslexia and statistical learning is still not fully understood. We contend that applying the multi-componential approach of statistical learning can provide a more precise picture of the statistical learning abilities of individuals with dyslexia. Indeed, looking at the literature from this multi-componential perspective suggests that performance on tasks that rely heavily on non-spatial distributional statistical learning (such as weather prediction tasks, cross-situational statistical learning tasks, or distributional category learning tasks) tends to reveal consistent impairments among individuals with dyslexia (Gabay, 2021; Gabay & Holt, 2015; Gabay et al., 2015a, b, 2023a, b; Kligler et al., 2023; Vandermosten et al., 2019; West et al., 2021). Similarly, deficits have also been observed in non-spatial conditional statistical learning tasks such as the triplet paradigm (Gabay et al., 2015a, b; Kligler & Gabay, 2023a; Ozernov-Palchik et al., 2023; Sigurdardottir et al., 2017; Singh et al., 2018), though one study failed to show such deficit (van Witteloostuijn et al., 2019). In the case of spatial statistical learning, the picture is more complex, and findings are not always consistent. In particular, performance on tasks that rely heavily on learning complex spatial configurations (e.g., contextual cuing tasks) has been reported to be intact in dyslexia (Howard Jr. et al., 2006; Jiménez-Fernández et al., 2011a; West et al., 2021), whereas on other tasks involving spatial horizontal regularities, such as serial reaction time tasks (Howard Jr. et al., 2006; West et al., 2021) or visual artificial grammar learning tasks (van Witteloostuijn et al., 2019), findings seem to be more mixed, regardless of structure (conditional vs. distributional).

### The present study

Overall, our review of the literature led us to predict that the statistical learning deficit in dyslexia may be more specific than previously assumed: It may involve a clear deficit in non-spatial statistical learning, but less impaired or even spared statistical learning abilities in the spatial domain. Based on prior research, this dissociation is expected to hold across both conditional and distributional forms of statistical structure. We focus on these aspects based on the theoretical analyses above as well as on recent evidence indicating that different tasks can tap into these related, yet distinct, components of statistical learning performance at the individual level (Growns et al., 2020). To examine our hypothesis, we investigated how individuals with dyslexia and cognitive- and age-matched neurotypicals detect and use these statistical regularities by employing a 2 × 2 factorial design that systematically manipulated both the type of statistical *relation* between elements (non-spatial vs. spatial) and the *structure* of statistical input (conditional vs. distributional).

## Methods

### Participants

The sample of participants consisted of two groups, a young adult dyslexia group (*N* = 31) and a neurotypical group (*N* = 30), with an age range of 18–37 years. All participants were native Hebrew speakers with no known hearing, neurological, linguistic, attentional, or intellectual impairments, and all were from families of middle to high socioeconomic status. The dyslexia group was mainly recruited through the Edmond J. Safra Brain Research Center for the Study of Learning Disabilities at the University of Haifa, Haifa, Israel. The presence of comorbid neurodevelopmental disorders such as attention-deficit hyperactivity disorder (ADHD), developmental language disorders, or any sensory or neurological disability constituted an exclusion criterion. Inclusion criteria for the dyslexia group were: (1) a formal diagnosis of dyslexia by a qualified psychologist; and (2) a score of at least one standard deviation below the mean for local norms on tests of phonological decoding (non-word reading). Since there are no standardized reading tests for adults in Hebrew, selection was based on local norms acquired from an independent sample, using criteria like those used in other studies conducted on Hebrew readers with dyslexia (Ballan et al., 2022; Kligler & Gabay, 2023a; Kligler et al., 2023). The decision to use scores of one standard deviation below the mean for local norms was based upon standard practice in the Hebrew literature (Breznitz & Misra, 2003; Shany & Breznitz, 2011). The control group consisted of participants who had no trouble with reading (e.g., at or above the inclusion criteria for the dyslexia group on the non-word reading test) and were at the same level of cognitive ability as the dyslexia group, as measured by the Raven test (Raven & Court, 1998).

This research was conducted in accordance with the Helsinki Declaration and approved by the Institutional Review Board at the University of Haifa. Participants provided written informed consent to participate in the study and received compensation for their involvement (150 shekels, approximately $40).

Participants underwent a series of cognitive tests designed to evaluate cognitive ability (Raven Test; Raven & Court, 1998), verbal short-term memory (Digit span; Wechsler et al., 2008), rapid automatized naming skills (RAN tests; Breznitz & Misra, 2003), phonological processing (phoneme segmentation, phoneme deletion, and spoonerism; Breznitz & Misra, 2003), word and non-word reading skills (Shatil, 1995, 1997), and attentional functions (Adult ADHD Self-Report Scale (ASRS); Konfortes, 2010). These tests were used to ascertain group differences in reading and phonological abilities. The results, shown in Table 1, indicate that the groups did not differ in age, cognitive abilities, or attentional skills. Additionally, the dyslexia group was significantly disadvantaged relative to the neurotypical group in reading ability (rate and accuracy measures of word reading and decoding skills), in phonological processing (rate and accuracy measures of phoneme segmentation, phoneme deletion, and spoonerism), and in rapid-naming skills, all of which are characteristics of developmental dyslexia, as well as in verbal short-term memory (digit span).

**Table 1:** Demographic and psychometric data of the dyslexia and control groups

Measurement	Control	*SD*	Dyslexia	*SD*	*t*-value	*p-*value
Age (in years)	25.233	2.699	25.871	3.836	0.748	0.457
Decoding						
Oral words recognition (accuracy)	110.333	14.965	64.483	21.410	−9.664	.001
Oral words recognition (speed)	110.967	14.879	67.807	22.728	−8.744	.001
Oral non-words recognition (accuracy)	62.533	8.885	28.677	10.772	−13.367	.001
Oral non-words recognition (speed)	65.933	9.425	41.968	12.518	−8.426	.001
Naming skills						
Naming letters (time)	21.933	2.391	25.709	4.181	4.347	.001
Naming objects (time)	33.5	6.19	40.742	8.029	3.936	.001
Naming numbers (time)	18.067	1.964	22.516	4.122	5.409	.001
Naming colors (time)	29.4	4.469	34.226	6.807	3.303	.002
Phonological processing						
Phoneme segmentation (time)	70.666	16.879	111.613	47.269	4.534	.001
Phoneme segmentation (accuracy)	14.9	1.213	12.193	3.390	−4.177	.001
Phoneme deletion (time)	98.967	19.98	184.807	62.859	−4.761	.001
Phoneme deletion (accuracy)	23.4	1.476	17.645	6.56	7.235	.001
Spoonerism (time)	109.233	17.338	258.58	118.709	6.929	.001
Spoonerism (accuracy)	19	1.287	14.807	4.11	−5.413	.001
Short verbal working memory						
Digit span	11.867	2.622	8.807	2.120	−5.02	.001
Intellectual ability						
Raven test	60.533	21.539	57.871	26.808	−0.427	0.671
Attentional functions						
Adult ADHD Self-Report Scale (ASRS)	33.1	6.402	36.032	9.368	1.431	0.158

### Statistical learning tasks

**Fig. 1:**
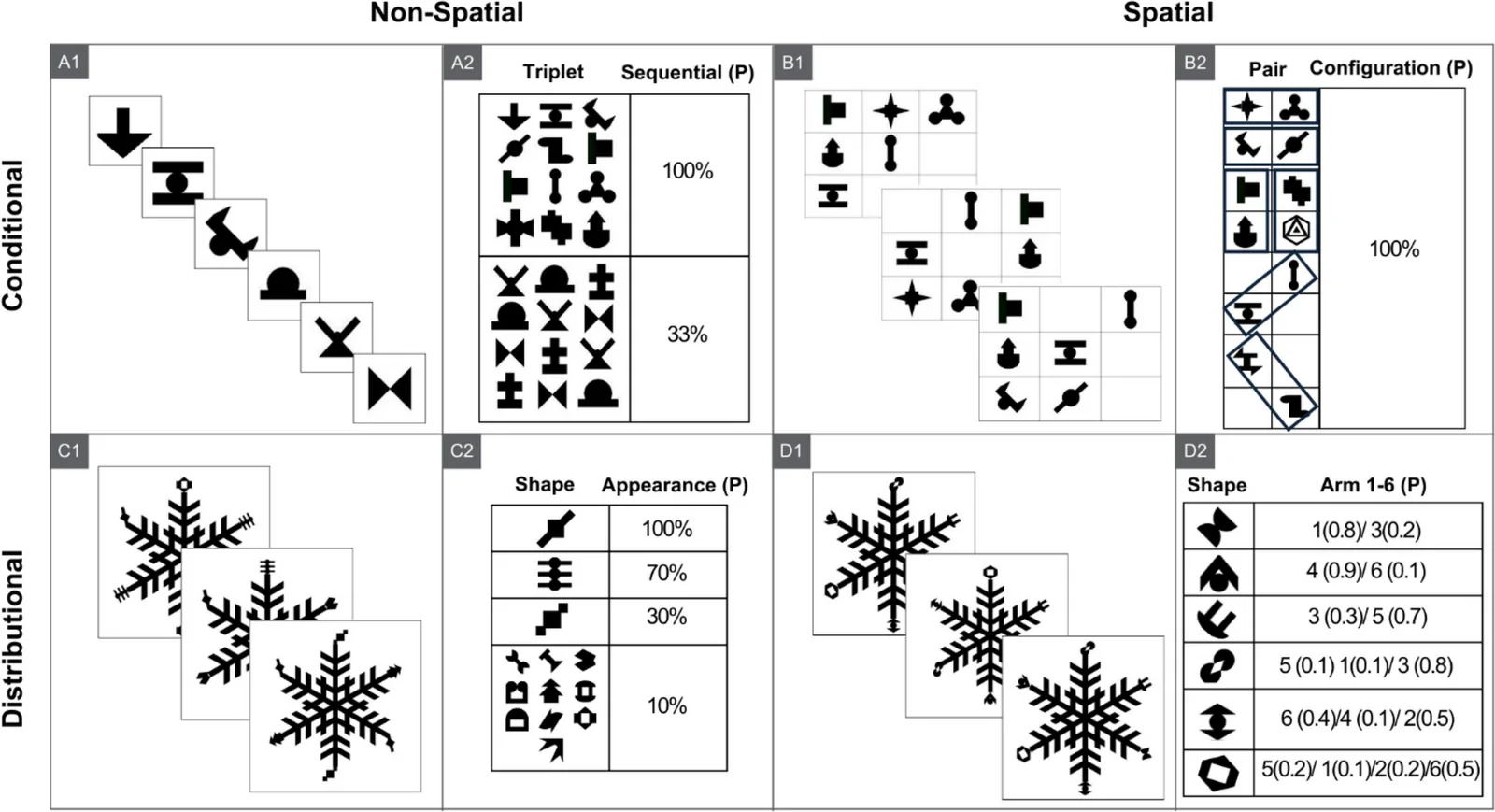
Overview of the four statistical learning tasks, their structure, relation type, and stimuli. **(A)** Non-spatial conditional statistical learning task. **(A1)** Example of a typical trial. **(A2)** Shapes comprising each triplet, with associated sequential probabilities. **(B)** Spatial conditional statistical learning task. **(B1)** Example of a typical trial. **(B2)** Stimulus pairs and their configural probability. **(C)** Non-spatial distributional statistical learning task. **(C1)** Example of a typical trial. **(C2)** Shapes and their appearance probabilities. **(D)** Spatial distributional statistical learning task. **(D1)** Example of a typical trial. **(D2)** Shapes and their probability of appearance across spatial locations (arms)

Following the methodology of Growns et al. (2020), our study employed four non-linguistic statistical learning tasks. Each task included an exposure phase and a test phase. During the exposure phase, participants observed stimuli structured according to predetermined statistical regularities. They were instructed to attend to the stimuli with the understanding that they would be asked questions about these stimuli later, but were not informed of the statistical regularities embedded in them. In the test phase, participants performed pattern recognition and pattern completion trials to assess their learning of the statistical regularities. In the pattern recognition trials, participants were presented with arrays of two to four shapes or shape patterns and were asked to identify the shape or pattern that appeared more familiar. In the pattern completion trials, participants selected the shape that “best completes the pair” in a sequence missing a target shape. To minimize interference across tasks, distinct sets of shapes were used in each task (see Fig. 1).

#### Non-spatial conditional statistical learning task

This task was originally developed by Siegelman et al., (2017a, b), and yields a reliable measure of statistical learning (Cronbach’s α =.88, split-half reliability =.83, and test–retest reliability =.68; Siegelman et al., 2017a, 2017b). The task began with a 10-min exposure phase in which participants were exposed to a temporally presented stream of visual stimuli composed of 16 individual shapes (see upper left panel of Fig. 1). Each shape was displayed for 800 ms, separated by a 200-ms interstimulus interval. The statistical structure of the stimuli was defined by their organization into eight predetermined triplets, which serve as the core units of conditional statistical regularity. The design includes two distinct categories of triplets. Four triplets, comprising a subset of four shapes, were characterized by transitional probabilities (TPs) of 0.33 (i.e., the four triplets 1–2–3, 2–1–4, 4–3-1, and 3–4-2, where each digit represents a shape). The remaining four triplets, formed from the remaining 12 shapes, had TPs of 1.0, indicating deterministic associations (e.g., 5–6–7). Each triplet was presented 24 times during the exposure phase, with randomized sequencing subject to the constraint that the same triplet cannot occur in consecutive presentations. Following exposure, participants proceeded to a test phase comprising 42 trials, subdivided into 34 pattern recognition trials and eight pattern completion trials. In the pattern recognition trials, participants were requested to discern the most familiar pair or triplet from a set of alternatives. These trials feature both target pairs/triplets derived from the exposure phase (with TPs of 0.33 or 1.0) and foil stimuli, which include sequences with TPs ranging from 0 to 0.50 and varying degrees of positional violations. Participants indicate their selections by pressing a computer key. In the pattern completion trials, participants were presented with a pair or triplet with one shape missing, and were instructed to select the shape “that best completes the pattern” from three options. This format assessed the participants’ ability to infer and extend the learned statistical structure. The sequence of pattern recognition and completion trials was randomized within each testing block to minimize procedural biases. Notably, chance performance in this task is quantified as 16.67 trials, comparable to an accuracy rate of 39.7%.

#### Spatial conditional statistical learning task

This task is based on the work of Fiser and Aslin (2001). In the exposure phase, participants viewed shape-pairs composed of 12 individual shapes (see upper right panel of Fig. 1). These shape-pairs were embedded within 144 3 × 3 grids. The grids were shown in a randomized sequence during a 7-min exposure phase. Each grid was displayed for 2,000 ms with no interstimulus interval (ISI). To manipulate conditional-spatial statistical information, shapes consistently appeared together within one of six predefined spatial pairings, each characterized by a spatial probability (SP) of 1. For instance, Shape 1 always co-occurred vertically above Shape 2, Shape 3 always appeared horizontally adjacent to Shape 4, and Shape 5 consistently co-occurred obliquely with Shape 6 (diagonal positioning). Across all trials, six shape-pairs were maintained (two vertical, two horizontal, and two oblique). Each grid contained six shapes, composed of three pairs, arranged so that each pair neighbored at least one other pair. The spatial relationships of the shape-pairs remained constant, allowing participants to extract pairings solely based on consistent spatial co-occurrences. In a test phase the participants completed 36 pattern recognition trials presented in a randomized sequence. On each trial, participants viewed two shape-pairs presented sequentially. They were asked to identify the shape-pair that seemed more familiar. The choices included shape-pairs from the exposure phase (SP = 1) and foil shape-pairs (SP = 0). Foil shape-pairs consisted of two shapes that had appeared in the tested positions of the grid during exposure but had never co-occurred in this specific spatial arrangement. Participants made their selections by pressing a computer key corresponding to their choice. Accuracy was determined by the SPs from the exposure phase, with chance-level performance set at 50% accuracy, equivalent to 18 correct trials.

#### Non-spatial distributional statistical learning task

This task was originally developed by Growns and Martire (2020), and provides a reliable measure of statistical learning (Cronbach’s α =.86 and split-half reliability =.76). Participants completed three blocks of exposure to a series of complex patterns composed of 13 individual shapes (see lower left panel of Fig. 1). These shapes appeared on “arms “ of 60 complex patterns (different snowflake figures). Across a 9-min exposure phase, patterns were presented for 3,000 ms each, separated by a 1,000-ms ISI. Participants were permitted brief, self-determined breaks between blocks. The presentation order of patterns was pseudorandomized in that all participants experienced the same sequence, which was generated randomly, whereas the statistical properties of the shapes’ distribution were manipulated. The appearance frequency of each shape varied systematically across the patterns viewed by the participant as follows: Shape 1 was present in all exemplars, Shape 2 in 30%, Shape 3 in 70%, and Shapes 4–13 in 10% of exemplars. These manipulations produced three joint probability categories for shape-pairs: 0.1 (0.1 × 1), 0.3 (0.3 × 1), and 0.7 (0.7 × 1). The entire set of 60 exemplars contained every possible spatial combination of shapes to ensure the distribution was independent of any spatial co-occurrences. Hence, statistical regularities are invariant to spatial positions. The statistically learned information relates to the identity of the shapes but not their location. As a result, Shapes 2 and 3 never co-occurred in the same exemplar. In a test phase, participants engaged in a total of 45 trials, comprising 33 pattern recognition trials and 12 nonsequential pattern completion trials, presented in a pseudorandomized sequence. During the pattern recognition trials, participants were asked to identify the most familiar shape or shape-pair from among two, three, or four options. Of these trials, 12 involved sequential presentation, where participants chose between two shapes displayed one after the other. The remaining 21 recognition trials required participants to choose among two, three, or four shape-pairs presented concurrently on the screen. In the pattern completion trials, participants selected the shape that “best completed the pair” from an array of two, three, or four shapes. Shapes presented during all trials were derived from exposure-phase frequencies ranging from 0.1 to 1, and shape-pairs were based on joint probabilities within a range of 0.1 to 0.7. The correct answer was based on the frequency or joint probabilities from the exposure phase. Participants indicated their choices by pressing designated computer keys, selecting either the most familiar shape/shape-pair or the shape completing the pair. Note that participants were instructed to ignore variations in shape orientation and spatial positioning when making their judgments. Performance at chance level for this task corresponded to 20.5 trials, equivalent to an accuracy rate of 45.5%.

#### Spatial distributional statistical learning task

This task was originally developed by Growns et al. (2020). During the exposure phase, participants completed three blocks, each lasting approximately 9 min. Within these blocks, they observed six distinct shapes presented on the “arms” of 60 complex patterns (see lower right panel of Fig. 1). These patterns followed a pseudorandomized order, with each individual pattern displayed for 3,000 ms, followed by a 1,000-ms ISI. Participants were permitted brief, self-determined breaks between blocks. The spatial distributions of the shapes were manipulated to embed distributional-spatial statistical information. Each shape exhibited unique probabilities of appearing on specific arms across all the patterns as follows: Shape 1 occurred with probabilities of 0.1 on Arm 1 and 0.9 on Arm 5. Shape 2 occurred with probabilities of 0.2 on Arm 4 and 0.8 on Arm 2. Shape 3 occurred with probabilities of 0.3 on Arm 3 and 0.7 on Arm 6. Shape 4 occurred with probabilities of 0.1 on Arm 5, 0.4 on Arm 1, and 0.5 on Arm 3. Shape 5 occurred with probabilities of 0.1 on Arms 2 and 6, 0.2 on Arm 1, and 0.6 on Arm 4. Shape 6 occurred with probabilities of 0.1 on Arm 2, 0.2 on Arms 3, 4, and 6, and 0.3 on Arm 1. The pseudorandomization of exemplars ensured that the statistical spatial distributions of the shapes were unconfounded by temporal regularities. Following the exposure phase, participants completed 45 test trials, including 26 pattern recognition trials and 19 pattern completion trials, presented in a pseudorandomized sequence. On pattern recognition trials, participants identified the most familiar shape in a specific spatial location. Arrays included two to four identical shapes positioned across different arms. The target shape appeared with the highest probability in a specific spatial location (e.g., 0.9 on Arm 1), while foils of the same shape occurred less frequently in other locations (e.g., 0.1 on Arm 5). On pattern completion trials, participants selected the shape and spatial location that “best completed the pair” from an array of shapes presented at various locations. Correct responses on these trials relied exclusively on the shape’s most frequent spatial location. Chance performance on the task was 16.62 trials, corresponding to 36.9% accuracy.

### Procedure

Initially, participants underwent comprehensive cognitive and linguistic assessments. They were then invited to the lab for an additional session during which they completed the four tasks in the following order: distributional non-spatial, conditional spatial, conditional non-spatial, and then distributional spatial, to minimize error variance across participants (Growns et al., 2020; Mollon et al., 2017).^1^ Most tasks used existing scripts and materials. Specifically, existing tasks created via Psychopy (Peirce, 2007) were used to run the non-spatial and spatial conditional statistical learning tasks. Similarly, an existing task created via Inquisit software (https://www.millisecond.com/) was used to run the non-spatial distributional learning task. The spatial distributional task was created according to the study by Growns et al. (2020) using E-prime Software (Schneider et al., 2002).

### Approach to analyses

#### Power analysis

We conducted a post hoc sensitivity analysis to estimate the smallest effect size detectable with 80% power at α = 0.05 given our sample size (*N* = 61). The analysis revealed we could reliably detect a small to medium effect size (*f* =.25) for the mixed-design ANOVA.

#### Statistical analysis

**Fig. 2:**
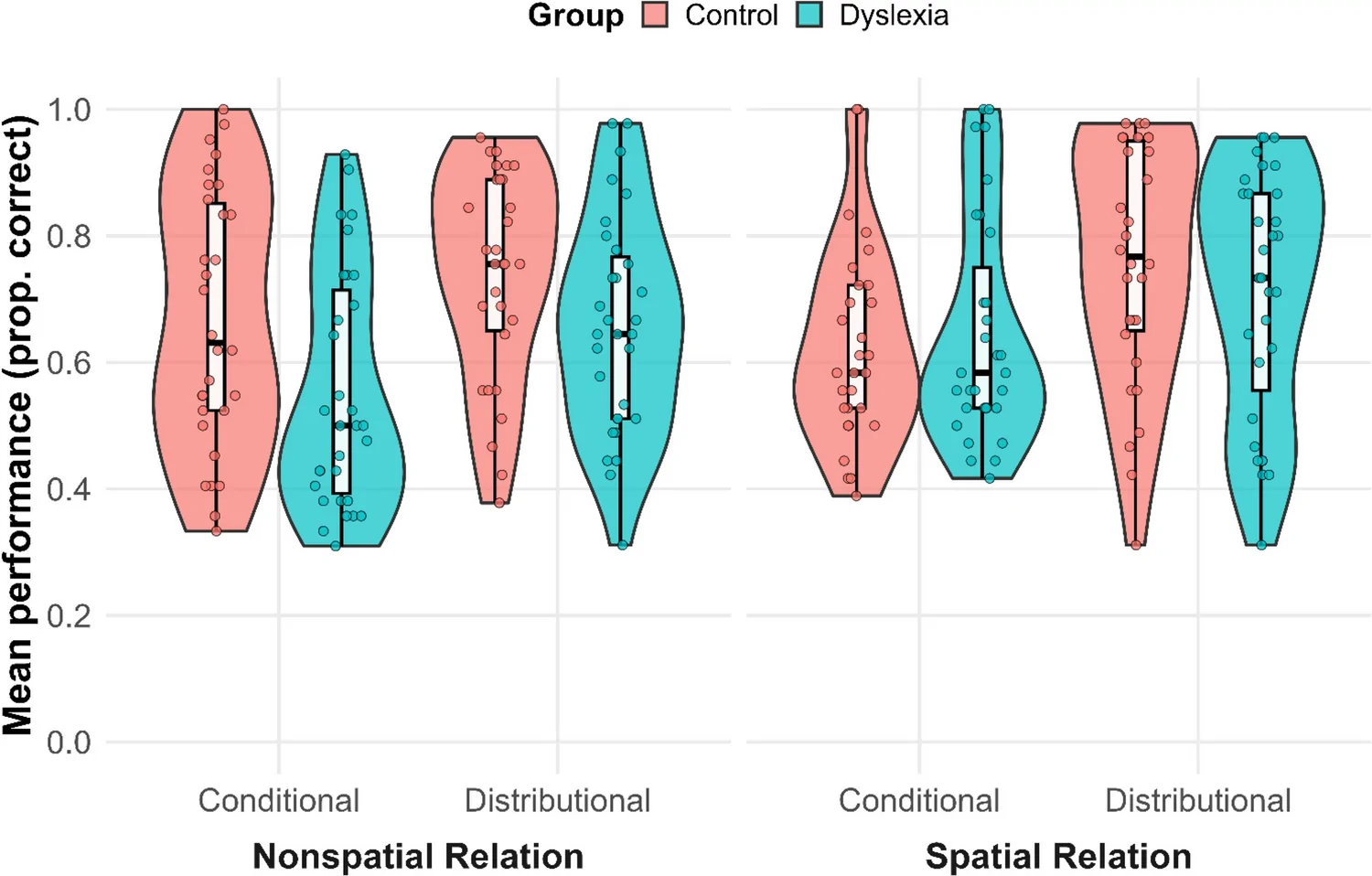
Statistical learning performance of the control and dyslexia groups as a function of relation type and statistical structure

First, we used one-sample *t*-tests to determine whether accuracy on each task occurred above chance level, with FDR (false discovery rate) correction for multiple comparisons. The purpose of this analysis was to determine whether significant learning occurred in each group. Table 2 shows the performance mean (and *SD*) for each task. Then, we used a generalized linear mixed-effects model (Jaeger, 2008), as implemented in the lme4 package (Bates et al., 2015) in R 4.1.2 (Core, 2015), to predict trial-level accuracy on each of the four tasks. Fixed effects were as follows: statistical structure type (transitional coded as 1, distributional coded as −1); relation type (non-spatial coded as 1, spatial coded as −1); group (dyslexia coded as 1, neurotypical coded as −1); and all interactions. The random-effects structure was composed of random intercepts for participants with random slopes for structure type and of relation type over all participants. Table 3 shows the results of this model, and Fig. 2 illustrates its effects. Finally, using the Bayesfactor package (Morey et al., 2015), Bayesian *t*-tests were conducted to explore the differences and similarities between groups. Finally, the relationships between cognitive and literacy measures and each of the statistical learning tasks were tested using simple Pearson correlations, see Table 4.

**Table 2:** Mean performance (and *SD*) of the control and dyslexia group in each statistical learning task

	Task	Group	Chance level	Mean_ACC	*SD*_ACC	*p*-value	FDR *p*-value
1	Non-spatial Distributional	DD	0.455	0.659	0.171	< 0.001	< 0.001
2	Non-spatial Distributional	TD	0.455	0.739	0.165	< 0.001	< 0.001
3	Non-spatial Conditional	DD	0.397	0.551	0.186	< 0.001	< 0.001
4	Non-spatial Conditional	TD	0.397	0.667	0.204	< 0.001	< 0.001
5	Spatial Distributional	DD	0.369	0.710	0.194	< 0.001	< 0.001
6	Spatial Distributional	TD	0.369	0.758	0.190	< 0.001	< 0.001
7	Spatial Conditional	DD	0.5	0.645	0.178	< 0.001	< 0.001
8	Spatial Conditional	TD	0.5	0.625	0.157	< 0.001	< 0.001

*ACC* accuracy, *FDR* false discovery rate, *DD* developmental dyslexia, *TD* control

**Table 3:** Results of the linear-mixed effects model

Correct				
Predictors	Odds ratios	Confidence interval	Statistic	*p*-value
(Intercept)	2.40	2.02–2.85	9.85	** < 0.001**
Diagnosis [TD]	1.17	0.99–1.40	1.79	0.073
learning type [Conditional]	0.77	0.69–0.87	−4.43	** < 0.001**
task type [Spatial]	1.10	1.00–1.21	1.94	0.052
trial Num	1.00	0.96–1.05	0.18	0.854
Diagnosis [TD] × learning type [Conditional]	0.97	0.86–1.08	−0.58	0.561
Diagnosis [TD] × task type [Spatial]	0.91	0.82–1.00	−2.03	**0.042**
learning type [Conditional] × task type [Spatial]	0.95	0.85–1.06	−0.91	0.362
(Diagnosis [TD] × learning type [Conditional]) × task type [Spatial]	0.92	0.83–1.02	−1.53	0.126

**Table 4:** Pearson correlations between performance in each of the statistical learning tasks and cognitive/literacy measures

Function	Measure	Non-spatial conditional	Non-spatial distributional	Spatial conditional	Spatial distributional
Decoding	Oral word recognition (accuracy)	.220	.137	-.022	-.040
	Oral word recognition (speed)	.237	.151	-.016	-.028
	Oral non-word recognition (accuracy)	.264*	.240	-.169	.188
	Oral non-word recognition (speed)	.228	.184	-.146	.057
Naming	Naming letters (time)	-.108	-.073	.174	.228
	Naming objects (time)	-.184	-.153	-.038	.069
	Naming numbers (time)	-.234	-.070	.077	-.036
	Naming colors (time)	-.146	-.265*	.054	.071
Phonological processing	Phoneme segmentation (time)	-.345**	-.341**	-.162	-.320*
	Phoneme segmentation (accuracy)	.215	-.064	-.022	.179
	Phoneme deletion (time)	-.351**	-.295*	-.111	-.288*
	Phoneme deletion (accuracy)	.238	.201	.015	.237
	Spoonerism (time)	-.302*	-.286*	-.083	-.305*
	Spoonerism (accuracy)	.241	.210	-.049	.149
Short verbal working memory	Digit span	.279^*^	.303^*^	.010	.009
Attentional functions	ASRS	-.074	-.205	-.103	-.066
Intellectual ability	Raven test	.110	.143	.095	.222

*ASRS* adult ADHD self-report scale

## Results

One simple *t*-test indicated that after FDR corrections (*p*s <.001) the control and dyslexia groups exhibited sensitivity to statistical input regularities on all tasks above the level of chance. A significant main effect emerged for structure type, with conditional statistics learned better than distributional statistics (Z = −4.39, *p* <.001). Importantly, the effect of relation type was modulated by group (Z = 2.03, *p* =.042).^2^ The Bayesian *t*-tests conducted indicated that individuals with dyslexia performed worse than controls when statistical regularities contained non-spatial relations (BF10 = 8.032279). On the other hand, the Bayes factor analysis supports no group differences when statistical regularities evoked spatial relations (BF01 = 4.725]. No other effects were significant. See also Online Supplementary Materials for the results of ANOVA analysis that mirror the results of the linear mixed-effects model.

## Discussion

Across four visual non-linguistic distinct statistical learning tasks, we assessed the ability of individuals with and without dyslexia to track conditional and distributional statistical structures with non-spatial or spatial relations during passive exposure. Results revealed that neurotypical learners demonstrate robust sensitivity to statistical regularities regardless of the statistical structure or the type of statistical relations, consistent with prior research (Growns et al., 2020). In contrast, learners with dyslexia exhibited a differential pattern, tracking statistical regularities embedded in spatial arrangements with a level of performance similar to that of neurotypicals, but struggling to learn regularities of a non-spatial nature. The impairment in sensitivity to non-spatial regularities was present in both conditional and distributional tasks, with performance exceeding chance level. These findings highlight specific constraints in statistical learning computations for individuals with dyslexia.

While some prior studies examined conditional or distributional statistical learning in dyslexia (e.g., Gabay & Holt, 2015; Kligler & Gabay, 2023b; Vandermosten et al., 2018) and others utilized spatial and non-spatial conditional statistical learning tasks (Bennett et al., 2008; Howard Jr. et al., 2006), no studies to date manipulated both aspects in the same experimental design. Our study therefore provides the first systematic examination of two major facets of statistical learning (type of relation between elements and structure of statistical regularities) using tasks that otherwise resemble each other, within the same individuals. This is important, as previous studies have used tasks that differ in multiple aspects (Bennett et al., 2008; Howard Jr. et al., 2006; West et al., 2018), which precludes a clear conclusion regarding statistical learning in dyslexia. This targeted methodological design then revealed that temporal aspects, specifically, are likely to contribute to the statistical learning deficit associated with dyslexia.

 Our findings highlight a more expansive non-spatial statistical learning deficit characteristic of dyslexia. Specifically, the *non-spatial* learning impairment in dyslexia is not confined to the learning of associations between successive events such as those embedded in common triplet statistical learning tasks (Kligler & Gabay, 2023a; Sigurdardottir et al., 2017). Rather, they encompass challenges in integrating non-spatially distributed information, both conditional and distributional. Difficulties in unsupervised linguistic *distributional* statistical learning have been previously reported among children with dyslexia (Vandermosten et al., 2019). Our findings suggest that the statistical learning gap is still apparent among high-functioning adults with dyslexia and is also present when non-linguistic stimuli are involved. More broadly, the observed deficit in non-spatial statistical learning among individuals with dyslexia and the robust associations between non-spatial statistical learning tasks and literacy-related skills (phonological processing, lexical retrieval, and phonological working memory; see also Table 4) lend further support for the involvement of non-spatial statistical learning computations in language acquisition, consistent with prior research (Arciuli & Simpson, 2012; Frost et al., 2013; Kligler & Gabay, 2023a; Qi et al., 2019; von Koss Torkildsen et al., 2019). Notably, robust associations were also observed for the spatial distributional learning task in which the performance of the dyslexia group did not differ significantly from that of controls. This pattern may suggest that spatial statistical learning is related to some components of literacy skills but does not differentiate between skilled and impaired readers.

Note that there are many differences in the tasks employed in the current study that might influence performance (duration, number of trials, etc.). However, the dissociation obtained in the dyslexia group was evident even though perceptual properties and task demands were similar in the spatial and non-spatial statistical learning tasks, in which the main difference was the type of information learned. In all tasks, learning took place and similar performance was observed in the spatial and non-spatial tasks among neurotypicals (see Fig. 2). This result shows that the spatial and non-spatial conditions did not differ in task demands or perceptual properties, indicating that these specific factors did not contribute to the group differences. Rather, the observed non-spatial statistical learning impairment in dyslexia may stem from a fundamental difficulty in aggregating statistical information across multiple experiences (Kligler et al., 2023). This interpretation aligns with the demands of the non-spatial tasks utilized in the current study, which require more extensive across-trial statistical computations compared to the spatial tasks. For instance, on the conditional spatial learning task, 50% of the information (three pairs) can be learned within a single trial. In contrast, the conditional non-spatial task necessitates at least three trials to learn just one of 16 triplets. Thus, an impaired ability to integrate statistical information across experiences can contribute to the challenge faced by individuals with dyslexia when learning non-spatial statistical relations. Alternatively, deficits in temporal learning and processing, often observed in dyslexia (Farmer & Klein, 1995; Gabay et al., 2012, 2019), may also contribute to the observed performance differences on the non-spatial tasks. Clarifying these possibilities will require further research. Future studies should aim to disentangle the contributions of temporal processing and statistical aggregation to the *non-spatial* statistical learning difficulties observed in dyslexia.

In this regard, observation of a non-linguistic statistical learning impairment extending beyond the linguistic domain provides evidence supporting the hypothesis that impairment in general learning mechanisms, not specific to language, can contribute to the challenges faced by individuals with dyslexia (Nicolson & Fawcett, 2019; Pennington et al., 2019; Ullman et al., 2020). In the context of understanding how dyslexia affects language processing, this evidence suggests that mechanisms affording the detection of non-spatial statistical regularities, such as those embedded in language, function less adequately among learners with dyslexia. Difficulties in the ability to utilize non-spatial statistical cues in support of speech comprehension (Holt, 2025) may influence the development of linguistic representations among learners with dyslexia. For example, the facilitation of phonological discrimination by non-spatial distributional learning (Maye et al., 2002) may be somewhat weaker in dyslexia (Gabay & Holt, 2015; Gabay et al., 2023a, b), leading to less robust phonological representations and hence phonological processing difficulties (Nicolson & Fawcett, 2019; Pennington et al., 2019). Difficulties in conditional non-spatial statistical learning could influence the ability of people with dyslexia to extract units from fluent speech, independent of language-specific knowledge. Further, such a deficit could influence the ability to detect visual co-occurrence of units in written language, thus compromising reading comprehension. Notably, although the tasks employed in the present study involve visual aspects, the statistical learning difficulties seen in dyslexia may represent a domain-general impairment and not necessarily a modality-specific deficit. We base our assumption on two observations: First, prior evidence suggests that auditory non-spatial conditional and distributional statistical learning computations are affected in dyslexia (Gabay, 2021; Gabay & Holt, 2015; Gabay et al., 2015a, b, 2023a, b; Vandermosten et al., 2019), leading us to believe that the difficulties seen in the present study are not specific to the visual modality. Second, this view is strengthened by the observation that in the present study performance on statistical learning tasks in the visual modality was correlated with performance in the auditory modality (e.g., phonological processing skills). This cross-modality relationship can be viewed as an indication of a domain-general deficiency. However, we also acknowledge that dyslexia is a heterogeneous phenomenon that might originate from different non-phonological deficits (Kristjánsson & Sigurdardottir, 2023; Share, 2021) and include several subtypes (Peterson et al., 2014), with phonological and surface dyslexia being the two main subtypes. Theories of phonological dyslexia state that the core deficit is access to the phonological lexicon, which is detrimental to acquisition of grapheme-phoneme mapping. On the other hand, surface dyslexia is claimed to involve an additional deficit in access to the orthographic lexicon. Future research should attempt to determine whether a particular subtype of dyslexia exhibits greater impairment in diverged statistical learning tasks as compared to others. Longitudinal studies will be most informative for revealing such patterns (Goswami, 2015).

The lack of an observed deficit in spatial statistical learning in individuals with dyslexia is also highly intriguing. Our study demonstrates that when statistical properties, such as conditional and distributional statistics, possess spatial rather than temporal relationships, individuals with dyslexia may exhibit spared performance on statistical learning tasks. The evidence in favor of the null hypothesis highlights that not all sorts of statistical learning are disrupted in dyslexia. Specifically, statistical learning processes rooted in the visuospatial domain may remain intact. Therefore, people with dyslexia may be able to capture spatial statistical learning relations incidentally through passive exposure. These observations align with prior evidence of intact and even enhanced visuospatial skills among learners with dyslexia (Von Karolyi et al., 2003), further underscoring the domain-specific nature of learning challenges associated with dyslexia. Therefore, the ability to detect spatial statistical regularities involved in written materials (Chetail, 2017; Lelonkiewicz et al., 2020; Siegelman et al., 2025) may be spared in dyslexia. Note, however, that some forms of spatial statistical learning computations are impaired in dyslexia (e.g., spatial statistical learning tasks involving horizontal regularities such as the serial reaction time task; Lum et al., 2013). Future studies are therefore needed to identify the nature of spatial statistical learning in dyslexia and its influence on reading performance. Furthermore, given the notion that orthographic depth across languages may entail fundamental disparities in the use of statistical learning when learning to read, future research should investigate whether the findings obtained in the present study are equally observed in different orthographies or logographic writing systems (e.g., Chinese) or if this finding is exclusive to opaque orthographies such as Hebrew (Shechter & Share, 2025).

The current findings also provide valuable insights into the underlying structure of statistical learning as a theoretical construct. Growns et al. (2020) already demonstrated that spatial and non-spatial statistical learning tasks are correlated, but not perfectly so. The same goes for transitional and distributional statistical learning tasks. Our findings contribute to this literature by showing that in a given special population (developmental dyslexia) specific deficits can be seen in one facet but not in another. Such domain-specific deficits are consistent with the multi-componential view of statistical learning (e.g., Arciuli, 2017; Bogaerts et al., 2022; Lukics & Lukács, 2022; Siegelman et al., 2017a, b), according to which there are *separable* (albeit not necessarily orthogonal) components to statistical learning, such that computations are impacted and constrained by the type of material or contingencies involved. Moreover, the robust associations observed between performance on specific statistical learning tasks and literacy-related skills further support this proposition.

In the broader context of NDDs, difficulties in sequential statistical learning despite intact non-sequential statistical learning have been associated with DLD (Gerken et al., 2021; Hsu & Bishop, 2014). Our findings of impaired performance in non-spatial statistical learning tasks (sequential and distributional) among people with dyslexia raise the possibility that people with DLD may also be impaired in non-spatial learning tasks in general and not specifically in sequential statistical learning. This is consistent with recent findings demonstrating impairments in non-spatial distributional statistical learning among children with DLD (Derawi et al., 2024). Given the involvement of statistical learning in language development and processing, it is important to consider how such an impaired ability may be manifested in diverse linguistic profiles of these two NDDs. One possibility is that both NDDs involve a deficit in statistical learning but differ in the severity of this impairment (DLD are impaired to a greater extent in statistical learning than dyslexia, a pattern that leads to additional language difficulties). Another possibility, which is consistent with multifactorial models of NDDs (Kouki et al., 2025), is that both populations suffer from shared cognitive deficits (e.g., statistical learning impairments) that are combined with unique cognitive impairments for each of the conditions (e.g., additional syntactic and semantic difficulties associated with DLD but not dyslexia). Lastly, a related possibility is that the nature of the statistical learning deficit differs between the two populations. For instance, both groups will show impairments in statistical learning processes related to phonological processing, whereas the DLD group may demonstrate additional statistical learning impairments in processes that may be spared or less impaired in dyslexia. Future studies are therefore required to reveal unique and shared properties of statistical learning impairments in dyslexia and DLD.

To summarize, the present study reveals a dissociation in the statistical learning abilities of young adults with dyslexia. Specifically, we observed impairments in their ability to extract and integrate statistical regularities with non-spatial relations, while their sensitivity to spatial statistical regularities remains intact. Moreover, statistical learning performance was correlated with linguistic skills. These findings suggest a specific difficulty in detecting and processing non-spatial statistical information over time, which may have implications for the development of linguistic representations in dyslexia. Further, the observed dissociation and the specific correlations found support the view that statistical learning is not a unitary construct but rather comprises distinct mechanisms for processing non-spatial and spatial regularities.

## Supplementary Information

Below is the link to the electronic supplementary material.

Supplementary file1 (DOCX 22 KB)

## Data Availability

The data and codes are available via the Open Science Framework at https://osf.io/5734e/.

## Footnotes

1 Counterbalancing the order of tasks has certain advantages, yet in this study we decided to keep the order constant to reduce variability between participants. As all participants performed the tasks in the same order, any effects of order should influence both groups similarly and hence group differences should represent differences in performance regardless of order.

2 When ASRS scores were added to the analysis as a covariate, the main results were still maintained.
