# Correction: The specific nature of the statistical learning deficit in developmental dyslexia: Impaired temporal and spared spatial learning abilities

**DOI:** 10.3758/s13423-026-03040-0

**Published:** 2026-11-02

**Authors:** Areen Mazzawi Shukri, Bethany Growns, Noam Siegelman, Yafit Gabay

**Affiliations:** 1 Department of Special Education and the Edmond J. Safra Brain Research Center for the Study of Learning Disabilities, University of Haifa, Mount Carmel, Haifa, Israel; 2 School of Psychological Sciences, Macquarie University, Sydney, NSW Australia; 3 Department of Psychology and Department of Cognitive and Brain Sciences, Hebrew University of Jerusalem, Jerusalem, Israel

**Correction: Psychonomic Bulletin & Review (2026) 33:238**

**https://doi.org/10.3758/s13423-026-02980-x**

The published version of the paper listed affiliations incorrectly. The correct attributions are below:

Areen Mazzawi Shukri^1^ · Bethany Growns^2^ · Noam Siegelman^3^* · Yafit Gabay^1^*

*Equal contribution

1 Department of Special Education and the Edmond J. Safra Brain Research Center for the Study of Learning Disabilities, University of Haifa, Israel

2 School of Psychological Sciences, Macquarie University, Sydney, NSW, Australia

3 Department of Psychology and Department of Cognitive and Brain Sciences, Hebrew University of Jerusalem, Jerusalem, Israel

