# Supplementary material for: The specific nature of the statistical learning deficit in developmental dyslexia: Impaired temporal and spared spatial learning abilities
Source: Psychon Bull Rev. 2026 Sep 15;33(7):238. doi: 10.3758/s13423-026-02980-x (PMC13577998; doi:10.3758/s13423-026-02980-x)
Supplement: Supplementary file 1 [file 13423_2026_2980_MOESM1_ESM.docx]

Supplemental materials, Mazzawi, Grown, Siegelman, & Gabay “Statistical Learning Performance in Individuals with Developmental Dyslexia: Evidence for Impaired Temporal and Spared Spatial Learning Abilities”, PBR

**Supplementary Table 1**

**Effects derived from Repeated Measures Analysis of Variance**

| **Effect** | **F** | **p** | **Partial eta-squared** |
| --- | --- | --- | --- |
| Group | 3.273 | .076 | .053 |
| Structure | 19.324 | .000 | .247 |
| Structure*Group | 0.123 | .727 | .002 |
| Relation | 2.899 | .094 | .047 |
| Relation*Group | 5.587 | .021 | .087 |
| Structure*Relation | 0.100 | .753 | .002 |
| Structure*Relation*Group | 1.638 | .206 | .027 |
